# Lipids at the meningeal-tumor interface: aging, anatomical niches, and meningioma biology

**DOI:** 10.3389/fnagi.2026.2005257

**Published:** 2026-10-29

**Authors:** Michele Cerasuolo, Nicholas Giulio Raccagni, Simone Laguardia, Gary Antonio Barahona Rodriguez

**Affiliations:** 1 Division of Human Anatomy, Neuronal Networks Morphology and Systems Biology Lab, Department of Mental and Physical Health and Preventive Medicine, University of Campania “Luigi Vanvitelli”, Naples, Italy; 2 Neuromodulation Center and Center for Clinical Research Learning, Spaulding Rehabilitation Hospital and Massachusetts General Hospital, Harvard Medical School, Boston, MA, United States; 3 School of Medicine and Surgery, University of Milano-Bicocca, Milan, Italy; 4 Beth Israel Deaconess Medical Center, Harvard Medical School, Boston, MA, United States

**Keywords:** aging, dura mater, lipidomics, meninges, meningioma, neuroinflammation, spatial lipidomics, tumor microenvironment

## Abstract

Meningiomas exhibit substantial molecular, anatomical, and microenvironmental heterogeneity, yet the spatial organization of their lipid biology remains poorly understood. Emerging lipidomic studies demonstrate extensive remodeling of membrane, signaling, and mitochondrial lipid classes, with profiles varying according to molecular genotype, histopathological grade, and potentially patient characteristics. In parallel, developmental, single-cell, and spatial studies have revealed the meninges and meningiomas as regionally organized systems comprising distinct stromal, vascular, immune, and neoplastic populations. Here, we integrate these lines of evidence and propose the aging meningeal lipid niche as an anatomically grounded framework for understanding lipid biology across the meningioma–meningeal interface. We examine the dural attachment and tumor–bone interface, perivascular niche, immune-enriched regions, tumor core, and tumor–brain boundary as candidate metabolic compartments in which cellular composition, vascular access, extracellular architecture, and inflammatory signaling may shape local lipid states. We further consider how aging may modify these environments through remodeling of the meningeal extracellular matrix, lymphatic function, immune composition, and lipid homeostasis, while distinguishing direct evidence in meningioma from mechanistic inference. A central limitation of current studies is their reliance on bulk tissue, which precludes confident attribution of lipid signals to specific anatomical and cellular sources. We therefore outline a spatially resolved strategy integrating anatomically registered sampling with mass spectrometry imaging, histopathology, molecular classification, and single-cell or spatial transcriptomic approaches. This framework provides testable hypotheses to determine whether meningioma lipid states reflect tumor-intrinsic programs, anatomically defined microenvironments, aging-associated remodeling, or interactions among these determinants, and how these states relate to invasion, recurrence, edema, and treatment resistance.

## 1 Introduction

Meningioma is the most frequently diagnosed primary intracranial tumor in adults and accounts for a substantial proportion of non-malignant tumors of the central nervous system (CNS). Its incidence increases markedly with age, providing an epidemiological rationale for considering meningioma in the context of the aging nervous system (Price et al., 2025). Although most are histologically low-grade and follow an indolent clinical course, they exhibit substantial biological and clinical heterogeneity. Recurrence, brain invasion, peritumoral edema, bone involvement, and resistance to radiotherapy occur across a spectrum only partially captured by conventional histopathological grading (Wang et al., 2024). Contemporary classification increasingly reflects this complexity: the 2021 WHO classification incorporated molecular criteria into meningioma grading, including oncogenic TERT promoter alterations and homozygous CDKN2A/B deletion as criteria for CNS WHO grade 3 (Louis et al., 2021). Subsequent cIMPACT-NOW recommendations have further refined molecular risk stratification, while molecular profiling has delineated biologically distinct meningioma groups with different oncogenic programs and clinical behavior (Sahm et al., 2025; Choudhury et al., 2022). Spatially resolved single-cell analyses further demonstrate that heterogeneity extends beyond the neoplastic compartment, revealing molecular-group-dependent differences in non-neoplastic cell states and dynamic changes across the transition from adjacent dura to tumor (Landry et al., 2026).

This molecular complexity must be considered within the distinctive anatomy of the tissues in which meningiomas develop. Rather than a uniform connective-tissue envelope, the meninges form a layered and regionally specialized interface linking the skull, CNS parenchyma, cerebrospinal fluid (CSF), vasculature, immune system, and peripheral lymphatic drainage (Betsholtz et al., 2024). Single-cell profiling of human dura further reveals heterogeneous stromal, vascular, and immune populations within the meningeal microenvironment (Wang et al., 2022). Anatomical connections among the dura, CSF, and calvarial bone marrow provide additional routes for molecular and cellular exchange across these compartments (Mazzitelli et al., 2022). Recent human meningeal development profiling further demonstrates layer-specific cellular programs and spatially organized neuroimmune interactions (Li Y. et al., 2026). These findings support a contemporary view of the meninges as a dynamic neurovascular–immune interface rather than as passive CNS coverings (Zhang et al., 2024). Developmental and molecular evidence also challenges the traditional assumption that all meningiomas arise from a homogeneous population of arachnoid cap cells, suggesting that cellular origin and local meningeal context may contribute to their anatomical and biological diversity (Pamir et al., 2026).

Lipid metabolism represents a comparatively underexplored dimension of this spatial biology. Lipids are integral to membrane organization, bioenergetics, intracellular and intercellular signaling, oxidative stress responses, and inflammation, all processes extensively remodeled by oncogenic and microenvironmental cues in cancer (Yu et al., 2021; Xiao et al., 2023). Direct evidence for lipid remodeling in meningioma has recently emerged. Lipidomic profiling of patient-matched meningioma and arachnoid membrane specimens shows alterations across glycerophospholipids, sphingolipids, cardiolipins, ceramides, and fatty-acid composition (Bogusiewicz et al., 2026). Complementary studies identified distinct lipidomic profiles associated with NF2 mutation status and histopathological grade (Halder et al., 2026). These findings indicate that lipid remodeling is heterogeneous in meningiomas and may vary with molecular background, pathological state, and patient characteristics. However, the existing studies predominantly rely on bulk-tissue measurements and cannot resolve the anatomical or cellular origins of individual lipid signals.

This topic is particularly timely given recent advances in meningioma lipidomics and single-cell and spatial profiling of meningeal and tumor tissues (Landry et al., 2026; Bogusiewicz et al., 2026; Li Y. et al., 2026). Beyond neuro-oncology, understanding lipid organization at the meningeal–tumor interface may contribute to broader questions in neuroscience concerning CNS border biology, neuroimmune communication, and age-related remodeling of the meningeal microenvironment.

This limitation becomes relevant with aging. Meningeal lymphatic function declines with age, impairing CNS fluid drainage and neuroimmune communication (Hitpass Romero et al., 2025), while age-related remodeling of the dural extracellular matrix (ECM) can further compromise lymphatic function and alter the immune environment (Hitpass Romero et al., 2025). In parallel, aging is accompanied by changes in CNS lipid homeostasis, including the emergence of lipid-droplet-accumulating, proinflammatory microglial states that link altered lipid handling to neuroimmune dysfunction (Marschallinger et al., 2020; Shao et al., 2026; Kim and Halliday, 2026). The convergence of age-dependent tissue remodeling, anatomical compartmentalization, and tumor variability raises a central question: are meningioma lipid states intrinsic properties of neoplastic cells, or do they emerge through interactions with the surrounding meningeal environment? We propose the aging meningeal lipid niche as an anatomically grounded framework where lipid states are interpreted across meningeal and tumor compartments and shaped by the intersection of cellular composition, molecular subtype, anatomical location, and vascular, immune, and age-related tissue features. By integrating evidence from meningioma lipidomics with meningeal anatomy, spatial biology, neuroinflammation, and brain aging, this review examines the spatial organization of lipid signaling across the meningioma–meningeal interface and outlines spatially resolved approaches to test this model.

## 2 Anatomical and biological organization of the meningeal–tumor interface

Although meningiomas are conventionally classified as extra-axial tumors, their anatomical position does not imply biological separation from the CNS. The meninges form an active interface in which vascular, stromal, immune, and lymphatic compartments interact with CSF and the brain surface (Betsholtz et al., 2024; Lohat et al., 2025; Delivanoglou et al., 2026). Dural lymphatic vessels provide routes for CNS fluid and macromolecular drainage toward peripheral lymphatic structures, while vascular channels connecting the dura and calvarial bone marrow enable local immune-cell trafficking (Hershenhouse et al., 2019; Herisson et al., 2018; El Kamouh et al., 2026). In contrast, the arachnoid barrier restricts exchange between the vascularized dura and CSF-containing subarachnoid compartment, whereas the inner arachnoid, pia, and associated perivascular structures interface closely with the cortical surface (Proulx and Engelhardt, 2022). Meningiomas therefore develop within a spatially organized tissue system exposed to diverse signals derived from the circulation, immune system, ECM, CSF, and adjacent nervous tissue.

This organization extends beyond the conventional division into dura, arachnoid, and pia. Single-cell profiling of adult human dura has identified heterogeneous fibroblast, endothelial, and immune populations, demonstrating substantial cellular diversity within the outer meningeal compartment (Wang et al., 2022). Complementary single-cell analysis of human leptomeninges has resolved distinct fibroblast, endothelial, mural, and immune populations, including arachnoid barrier-associated and perivascular fibroblast states (Kearns et al., 2023). The dura is particularly complex: its collagen-rich matrix contains vascular, lymphatic, stromal, neural, and immune components and maintains direct communication with calvarial bone marrow through transosseous channels (Mazzitelli et al., 2022). Because these compartmental differences create distinct biochemical environments, they are likely to shape local exposure to circulating metabolites and lipoproteins, oxygen, CSF-derived molecules, and inflammatory mediators.

Aging remodels several components of this system, progressively altering the structural and immunological context of the meningeal environment (Yuan et al., 2026). Meningeal lymphatic function declines with age, impairing CNS fluid drainage and neuroimmune communication and potentially modifying the clearance of metabolites and inflammatory mediators from meningeal compartments (Elyse Moore et al., 2026). Age-associated remodeling of the dural ECM, including peri-lymphatic collagen accumulation driven in part by immune–fibroblast signaling, can further disrupt lymphatic integrity, lymphangiogenic capacity, and CSF clearance (Hitpass Romero et al., 2025). In parallel, aging alters meningeal immunity, with T-cell accumulation and increased IFNγ signaling contributing experimentally to lymphatic dysfunction and illustrating the functional coupling between immune and lymphatic changes at the aging CNS border (Rustenhoven et al., 2023). More broadly, age-related changes in meningeal cellular composition, vascular organization, and tissue homeostasis provide a biological basis for considering age as a modifier of the local environment in which meningiomas develop, rather than solely as an epidemiological variable (Chirica et al., 2026). Whether these age-associated changes directly influence meningioma biology remains unresolved.

The biological relevance of anatomy is reinforced by associations between meningioma location and the molecular background. NF2-altered meningiomas are enriched at the cerebral convexities and other non-midline locations, while non-NF2 drivers, including AKT1, SMO, PIK3CA, TRAF7, and KLF4, show preferential distributions across skull-base territories (Clark et al., 2013). More recent molecular profiling has confirmed significant associations between tumor location and genotype, with NF2 alterations predominating in convexity tumors and non-NF2 alterations enriched in lower-grade skull-base meningiomas (Ali et al., 2025). These relationships persist at finer anatomical scales: within the foramen magnum, TRAF7 and AKT1 alterations are preferentially associated with anterolateral tumors, while NF2 alterations are enriched in posterior lesions (Hua et al., 2024). Although not absolute, these associations indicate that anatomical location is linked to molecular variability and may reflect the intersection of developmental lineage, local tissue architecture, microenvironmental exposure, and tumor genotype.

These anatomical, molecular, and age-dependent differences provide a rationale for examining lipid signaling spatially. Vascularized dural compartments have access to circulating lipids and lipoproteins and contain metabolically active endothelial, stromal, and immune populations, whereas CSF-facing compartments are subject to distinct transport constraints and extracellular environments. Matrix-rich dural attachments, perivascular regions, and cortical interfaces may consequently differ in lipid uptake and turnover, lipid-mediator production, and oxidative conditions. Because meningioma specimens contain variable proportions of neoplastic, immune, vascular, and stromal cells, bulk lipidomic measurements may integrate signals from multiple cellular and anatomical sources. Existing studies demonstrate remodeling across phospholipid, sphingolipid, cardiolipin, ceramide, and fatty-acid metabolism, but do not establish how these alterations are distributed across anatomical or cellular compartments. Anatomically distinct lipid niches should therefore be regarded as a testable spatial model rather than an established metabolic classification of meningioma.

## 3 Developmental origin and spatial cellular identity of meningioma

The traditional model of meningioma histogenesis has centered on arachnoid cap cells, based largely on morphological similarities between meningothelial tumor cells and arachnoid-derived structures. This model, however, is increasingly difficult to reconcile with the developmental and molecular heterogeneity of the meninges. Cranial meningeal tissues do not arise from a single embryological lineage: neural crest and cephalic mesoderm contribute differentially across anatomical territories, generating regionally distinct meningeal compartments with distinct developmental trajectories (Dasgupta and Jeong, 2019). Molecular studies provide a potential link between this developmental topography and meningioma biology, with specific driver alterations showing preferential distributions across meningeal territories assigned to different embryological origins (Boetto et al., 2021). Recent spatiotemporal profiling of the developing human meninges further demonstrates that meningeal layers acquire distinct cellular and transcriptional identities during gestation, including layer-specific stromal states, immune distributions, barrier-associated programs, and metabolic features (Li Y. et al., 2026). A complementary multiomic study integrating single-nucleus RNA sequencing, chromatin accessibility, and spatial transcriptomics further characterized endothelial barrier regulatory programs and identified potential chemotactic interactions between cranial marrow and the developing meninges (To et al., 2026). Taken together, these observations support a regionally specified model of meningeal development and provide a developmental basis for reconsidering a universally uniform arachnoid cell of origin for meningioma.

Consistent with this framework, recent synthesis of developmental, molecular, single-cell, and experimental evidence has challenged an exclusively arachnoid cap-cell origin and raised the possibility that meningiomas may arise from regionally distinct progenitors (Pamir et al., 2026). A recent single-cell and spatial transcriptomic atlas of human fetal meninges identified an inner CDH1-positive dural population with barrier-associated features. Comparative analyses revealed greater transcriptional similarity between meningioma cells and dura-lineage populations than between meningioma cells and arachnoid populations, alongside expression of common meningioma driver genes in the dural lineage (Vinsland et al., 2026). These findings support a possible dura-lineage origin for at least a subset of meningiomas, although transcriptional similarity does not establish cellular origin and this hypothesis requires further functional validation.

Developmental origin is unlikely to operate independently of subsequent tumor evolution. Spatially resolved single-cell profiling demonstrates that meningioma cells coexist with heterogeneous immune, vascular, and stromal populations whose abundance and transcriptional states vary across molecular groups and the dura–tumor interface, with particularly pronounced spatial reprogramming of myeloid populations and state-specific myeloid–neoplastic interactions (Landry et al., 2026). Intratumoral spatial profiling further reveals regional divergence in gene and protein expression and spatial expansion of subclonal copy-number alterations associated with recurrence and treatment resistance (Bankole et al., 2026). These findings support a model of meningioma as a heterogeneous ecosystem in which developmental lineage and tumor genotype intersect with microenvironmental context and acquired cellular states.

This framework has key implications for interpreting meningioma lipid biology. Developmental lineage and cellular identity may shape membrane organization, receptor signaling, metabolic enzyme expression, and interactions with extracellular and vascular substrates, while spatially distinct immune, vascular, and stromal populations may provide additional sources and modifiers of tissue lipid signals. Consequently, lipid signatures detected in bulk meningioma may reflect not only tumor-cell-intrinsic metabolism but also local substrate availability, non-neoplastic cellular composition, and interactions among these determinants. A central unresolved question is whether meningioma lipid states are lineage-linked tumor-intrinsic programs, adaptive responses to anatomically distinct microenvironments, or combinations of both. Resolving these possibilities will require integrating developmental and molecular identity with spatially registered lipidomics and cellular profiling.

## 4 Lipid remodeling in meningioma: evidence and molecular determinants

Evidence for lipid remodeling in meningioma has progressed from mass-spectrometric molecular fingerprints to comprehensive lipidomic analyses linking lipid composition with tumor genotype, histopathological grade, and patient characteristics. Early desorption electrospray ionization mass-spectrometry imaging (DESI-MSI) demonstrated distinct lipid-associated molecular profiles in meningioma and adjacent dura, discriminated fibroblastic from meningothelial tumors, and identified molecular patterns associated with NF2 aberrations (Calligaris et al., 2015). These findings provided early evidence that lipid-associated molecular signatures capture both tumor identity and biological heterogeneity while showing the potential of mass-spectrometry imaging to resolve within-tissue differences.

More direct evidence comes from comprehensive lipidomic analysis of patient-matched meningioma and arachnoid membrane specimens. Chen et al. (2026) identified increased total lipid abundance in tumor tissue accompanied by extensive remodeling of glycerophospholipids, sphingolipids, cardiolipins, ceramides, and fatty acids; PC, PE, and CL were increased, whereas PI was reduced, fatty-acid composition shifted toward lower saturated and higher polyunsaturated species, and selected methylphosphatidylcholine- and sphingomyelin-related measures were associated with tumor size or patient age. These findings extend earlier biochemical evidence of altered phospholipid composition and increased fatty-acid unsaturation in meningioma relative to normal leptomeninges (Riboni et al., 1984). The observed cardiolipin changes are consistent with remodeling of mitochondrial membranes, given the central role of cardiolipin in inner mitochondrial membrane organization and bioenergetic function, but do not establish mitochondrial biogenesis or altered respiratory activity (Paradies et al., 2019). Likewise, enrichment of polyunsaturated fatty acids may influence membrane organization and signaling while increasing the availability of substrates susceptible to lipid peroxidation (Ayala et al., 2014). The paired design reduces interindividual variability and provides an anatomically relevant meningeal comparator; but the small cohort and bulk-tissue approach preclude cellular attribution and mechanistic inference.

Lipid composition also appears to vary with tumor genotype. Using solid-phase microextraction coupled with liquid chromatography–high-resolution mass spectrometry, Bogusiewicz et al. (2026) identified 34 lipids contributing to the discrimination of NF2-mutant from NF2-wild-type meningiomas, spanning glycerophospholipids, sphingolipids, hexosylceramides, and related species. Notably, complementary targeted profiling demonstrated higher concentrations of short-, medium-, and long-chain acylcarnitines in NF2-mutant meningiomas, with 10 of 15 detected species differing significantly according to NF2 status (Bogusiewicz et al., 2025). Because acylcarnitines participate in mitochondrial fatty-acid transport and oxidation, these findings suggest an association between NF2 status and mitochondrial lipid handling; however, their accumulation may also reflect incomplete β-oxidation or an imbalance between fatty-acid delivery and mitochondrial oxidative capacity and therefore does not establish increased fatty-acid oxidation (Bjørndal et al., 2018). Direct metabolic-flux studies will be required to determine whether these genotype-associated lipid profiles reflect altered fatty-acid oxidation, mitochondrial metabolic capacity, or other metabolic consequences of NF2 loss.

Experimental evidence also implicates fatty acid synthase (FASN), a key enzyme in *de novo* fatty acid synthesis, in meningioma growth. Haase et al. reported increased FASN expression in higher-grade meningiomas compared with grade I tumors. Pharmacological inhibition with cerulenin reduced meningioma-cell survival *in vitro* and tumor growth in xenograft models, accompanied by decreased AKT phosphorylation. In addition, inhibition of PI3K signaling reduced FASN expression, suggesting a functional relationship between PI3K/AKT signaling and fatty acid synthesis in meningioma cells (Haase et al., 2010). These findings provide a mechanistic link between lipid metabolism and tumor growth, although clinical validation remains lacking.

Cholesterol biosynthesis represents another lipid metabolic pathway implicated in meningioma progression. Li H. et al. (2026) demonstrated that 3-hydroxy-3-methylglutaryl-CoA reductase (HMGCR), the rate-limiting enzyme of cholesterol biosynthesis, contributes to meningioma-cell proliferation and invasion. HMGCR silencing reduced intracellular cholesterol levels and PI3K/AKT phosphorylation, while pharmacological AKT activation partially reversed the inhibitory effects on tumor-cell growth and invasion. The authors also showed that brusatol suppressed meningioma progression in experimental models through HMGCR downregulation and inhibition of PI3K/AKT signaling. These findings suggest a functional relationship between cholesterol metabolism and oncogenic signaling in meningioma, although clinical validation remains necessary.

Histopathological grade is another axis of lipid heterogeneity. Untargeted LC-HRMS profiling of 85 meningioma biopsies identified metabolomic and lipidomic differences between lower- and higher-grade tumors, with feature selection and machine-learning approaches identifying a small set of molecular features that discriminated these groups within the study cohort (Safari Yazd et al., 2023). More recently, integrated tissue metabolomics and lipidomics, complemented by serum lipidomics, revealed grade-associated alterations in choline, sphingolipid, and glycerophospholipid metabolism, with PC and PE species comprising a substantial proportion of altered lipids and additional differences in carbon-chain length and saturation; an 18-lipid panel was identified as potentially discriminatory between lower- and higher-grade tumors (Halder et al., 2026). Together, these studies support an association between lipid composition and histopathological grade, but differences in cohort composition, analytical platforms, biospecimen type, grading frameworks, and statistical feature selection, together with the absence of external validation, currently preclude definition of a reproducible grade-specific lipid signature.

Sphingolipid remodeling emerges as a recurrent feature of meningioma, although without a consistent pattern. Alterations involving sphingomyelins, ceramides, sphinganine, and hexosylceramides vary across experimental comparisons and molecular species. This heterogeneity may be biologically relevant because sphingolipids function as both membrane constituents and bioactive mediators of cellular stress, inflammation, proliferation, and cell-death signaling, with their effects shaped by molecular species, metabolic interconversion, subcellular localization, and cellular context (Kuo and Hla, 2024). Current evidence therefore supports context-dependent reorganization of sphingolipid homeostasis rather than a uniform pro-survival or pro-death state. Similar caution applies to plasmalogens, neutral lipids, cholesterol-related species, and other lipid pools for which meningioma-associated differences have been reported but whose cellular origins and functional consequences remain unresolved.

Taken together, current evidence establishes lipid remodeling as a recurrent feature of meningioma but does not support a single, uniform lipid phenotype. Lipid profiles vary with the tissue comparator, molecular genotype, histopathological grade, and potentially patient characteristics such as age, indicating that meningioma lipid biology is context-dependent. The principal unresolved limitation is spatial and cellular attribution: bulk-tissue measurements integrate neoplastic, immune, vascular, and stromal compartments, while mass-spectrometry imaging can localize lipid-associated signals within tissue but does not by itself identify their cellular sources or the metabolic processes that generate them. Consequently, observed lipid alterations cannot yet be confidently assigned to tumor-cell-intrinsic metabolism, specific components of the meningeal microenvironment, or interactions between them. Resolving this distinction will require moving beyond whole-tumor lipid profiling toward anatomically registered, spatially resolved analysis of the tumor–meningeal interface. Taken together, current evidence establishes lipid remodeling as a recurrent feature of meningioma but does not support a single, uniform lipid phenotype (Table 1).

**Table 1:** Human studies characterizing lipid remodeling in meningioma.

Study	Cohort/Comparison	Platform	Lipid domain(s)	Principal findings	Biological correlate
Riboni et al. (1984)	Meningioma vs. normal leptomeninges	Biochemical lipid analysis	Phospholipids; fatty acids	Altered phospholipid composition; ↑fatty-acid unsaturation	Tumor tissue
Calligaris et al. (2015)	Meningioma vs. dura; tumor subgroups	DESI-MSI	Lipid-associated molecular profiles	Spatial molecular profiles distinguished tumor from dura and fibroblastic from meningothelial tumors	Histology; NF2 aberration
Safari Yazd et al. (2023)	85 biopsies; lower vs. higher grade	LC-HRMS	Metabolomic and lipidomic features	Grade-associated molecular profiles and discriminatory features	WHO grade
Bogusiewicz et al. (2025)	NF2-mutant vs. NF2-WT	Targeted profiling	Acylcarnitines	↑Short-, medium-, and long-chain acylcarnitines in NF2-mutant tumors; 10/15 species differed	NF2 status
Chen et al. (2026)	12 paired meningioma–arachnoid specimens	Tissue lipidomics	GP; SP; CL; Cer; FA	↑Total lipids, PC, PE and CL; ↓ PI; ↓ saturated and ↑ polyunsaturated FA; broader SP/Cer remodeling	Tumor vs. arachnoid; exploratory age/size associations
Bogusiewicz et al. (2026)	NF2-mutant vs. NF2-WT	SPME–LC-HRMS	GP; SP; HexCer; related lipids	34 discriminatory lipid species; genotype-associated species-level remodeling	NF2 status
Halder et al. (2026)	Tissue ± serum; lower vs. higher grade	Integrated metabolomics/lipidomics	PC; PE; SP; choline-related pathways	Grade-associated lipid remodeling; differences in chain length/saturation; 18-lipid discriminatory panel	WHO grade

Cer, ceramide; CL, cardiolipin; DESI-MSI, desorption electrospray ionization mass spectrometry imaging; FA, fatty acid; GP, glycerophospholipid; HexCer, hexosylceramide; LC-HRMS, liquid chromatography–high-resolution mass spectrometry; PC, phosphatidylcholine; PE, phosphatidylethanolamine; PI, phosphatidylinositol; SP, sphingolipid; SPME, solid-phase microextraction; WT, wild type. ↑ and ↓ indicate higher and lower abundance, respectively, in the first group relative to the stated comparator. Studies differ substantially in analytical platform, biospecimen, comparator, and statistical methodology; discriminatory signatures should therefore not be interpreted as a validated common meningioma lipid signature.

## 5 Spatial lipid niches across the meningioma microenvironment

The current evidence landscape highlights a marked gap between lipid remodeling demonstrated in bulk meningioma tissue and its spatial attribution across anatomically distinct tumor–meningeal compartments (Figure 1). These compartments are associated with distinct biological processes, including extracellular matrix and bone remodeling, angiogenesis and vascular regulation, immune-cell recruitment and inflammatory signaling, tumor-cell proliferation, and glial responses and invasion at the tumor–brain interface.

**Figure 1:**
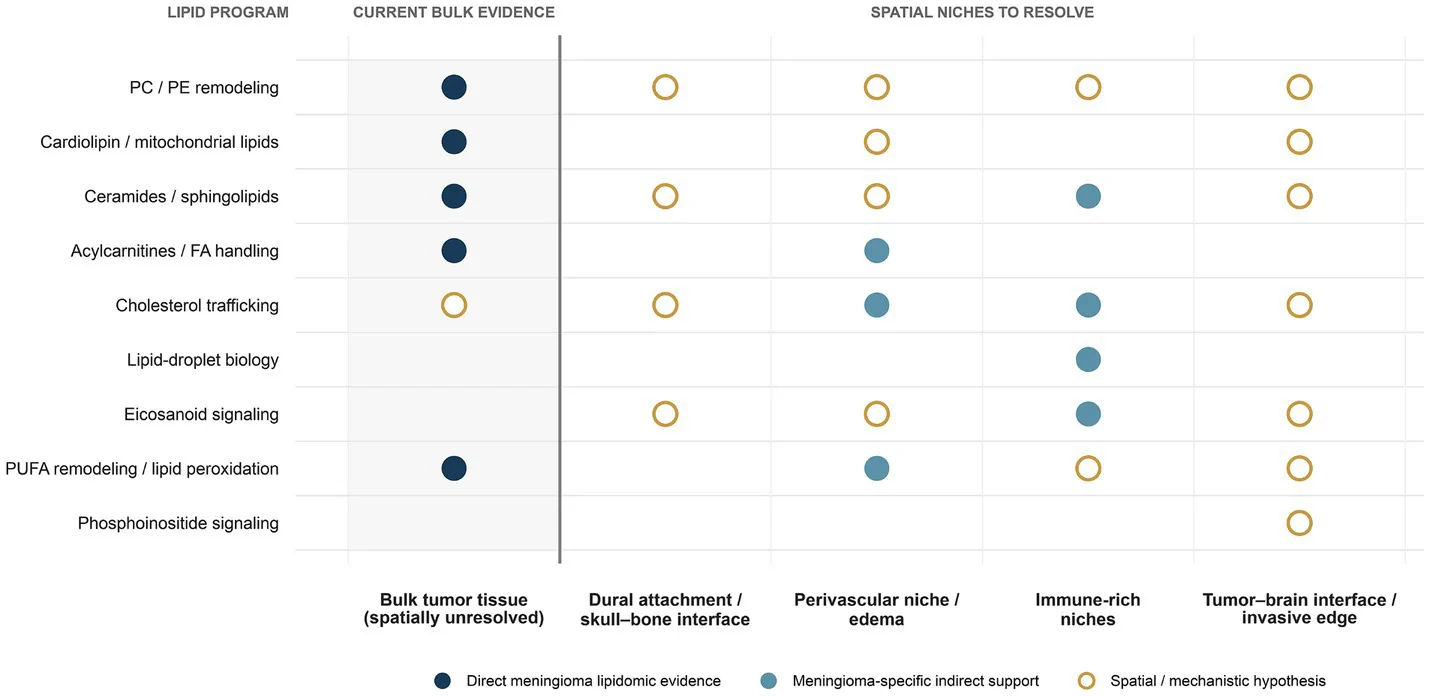
Spatial lipid evidence landscape across the meningioma–meningeal interface. The matrix summarizes the current level and spatial specificity of evidence for major lipid programs in bulk meningioma tissue and across candidate anatomical niches. Dark blue circles indicate direct meningioma lipidomic evidence; light blue circles indicate meningioma-specific indirect support from anatomical, cellular, vascular, or immune studies in the absence of direct compartment-resolved lipid measurements; and open circles indicate spatial or mechanistic hypotheses that remain to be experimentally tested. Candidate compartments include the dural attachment and skull–bone interface, perivascular and edema-associated regions, immune-rich niches, and the tumor–brain interface or invasive edge. Evidence categories indicate the level and spatial specificity of current support and should not be interpreted as measures of effect size or biological importance.

### 5.1 Dural attachment and skull–bone interface

The dural attachment is the defining anatomical anchor of most meningiomas and constitutes a biologically complex interface rather than merely a surgical boundary. The dura is a collagen-rich, highly vascularized tissue containing heterogeneous cellular populations and is directly apposed to the inner surface of the skull (Neish et al., 2026). Transosseous vascular channels connect calvarial bone marrow with the dura, providing anatomical routes for local immune-cell trafficking and communication between these contiguous compartments (Liu et al., 2025). The calvarial marrow itself represents a specialized hematopoietic niche functionally coupled to CNS-border immunity, further integrating the skull and meninges within a shared neuroimmune system (Koh and Adams, 2026). In meningioma, this anatomical continuity creates a tumor–dura–bone interface in which neoplastic cells encounter stromal, vascular, lymphatic, and immune populations that may differ substantially from those within the tumor core.

The lipid biology of this interface remains largely unexplored. Hyperostosis and osseous invasion reflect substantial remodeling at the tumor–bone boundary, although these processes are biologically heterogeneous and should not be considered interchangeable manifestations of bone involvement (Cook et al., 2026). This heterogeneity raises the testable possibility that the tumor–dura–bone interface harbors a lipid state distinct from those of the tumor core and uninvolved dura. Testing this hypothesis will require anatomically registered sampling of the tumor core, dural attachment, hyperostotic or invaded bone, and uninvolved adjacent dura as discrete compartments. Integrating mass-spectrometry imaging with histopathology, molecular classification, and spatial or single-cell profiling could then determine whether compartment-specific lipid states are associated with matrix remodeling, osteogenic programs, vascular organization, immune-cell composition, or local tumor invasion.

### 5.2 Perivascular niche and edema

The perivascular compartment provides a compelling example of spatially restricted biology within meningioma. Choudhury et al. (2024) identified perivascular NOTCH3-positive stem-like cells conserved across human, canine, and experimental meningiomas and demonstrated that NOTCH3 promotes tumor-initiating capacity, proliferation, angiogenesis, and radiotherapy resistance, establishing the vascular niche as a functionally distinct neoplastic compartment. Beyond its organization, this niche provides access to circulating lipids and lipoproteins, oxygen, and inflammatory signals, while endothelial and mural cells may further shape local substrate availability and paracrine signaling. Whether these features generate a distinct perivascular lipid state in meningioma remains unknown.

Recent experimental evidence links NOTCH3 signaling to fatty acid metabolic reprogramming in meningioma. Sadagopan et al. demonstrated that NOTCH3-expressing meningioma cells exhibit increased expression of CD36, a fatty acid transporter, and enhanced mitochondrial fatty acid oxidation. NOTCH3 overexpression was associated with transcriptional upregulation of CPT1A, CPT2, and HADHA, enzymes involved in mitochondrial fatty acid oxidation, together with reduced cellular fatty acid pools. These metabolic changes were accompanied by increased resistance to ferroptosis, which was reversed by pharmacological inhibition of CPT1 (Sadagopan et al., 2025). These findings identify a potential mechanism linking NOTCH3 signaling, lipid utilization, and tumor-cell survival. However, whether this metabolic program is specifically enriched within the perivascular niche of human meningiomas remains to be established.

Peritumoral brain edema provides a clinically measurable manifestation of interactions among meningioma, its vasculature, and the adjacent brain. Its pathogenesis is multifactorial, with evidence implicating VEGF-dependent vascular permeability, matrix metalloproteinases, aquaporins, inflammatory mediators, mast cells, and disruption of tissue and vascular barriers (Zhang et al., 2026). Lipid signaling provides a plausible but largely unexplored link to these processes because bioactive lipid mediators can regulate endothelial permeability, vascular tone, leukocyte recruitment, and inflammatory responses (Chistyakov et al., 2025). However, direct evidence connecting specific lipid pathways to meningioma-associated edema is currently lacking. Spatially registered analysis of perivascular and non-perivascular tumor regions could therefore determine whether phospholipid, sphingolipid, oxidized-lipid, or eicosanoid profiles vary with vessel density, NOTCH3-positive tumor states, vascular-barrier features, and MRI-defined edema. Such studies would help distinguish lipid programs associated with vascular-niche biology from tumor-wide metabolic remodeling.

### 5.3 Immune-rich niches

Immune composition represents another major source of spatial and metabolic heterogeneity. Multimodal profiling of 2,727 meningiomas using single-cell RNA sequencing, mass cytometry, bulk transcriptomics, DNA methylation, and multiplex imaging showed extensive immune infiltration, with macrophages constituting the predominant immune population and their differentiation states varying with tumor grade, recurrence, and metastatic progression (Guo et al., 2026). These findings argue against treating the immune infiltrate as a uniform background component and instead support biologically distinct immune states that may contribute differently to the local tumor microenvironment (Youngblood et al., 2026).

Lipid metabolism is closely coupled to macrophage functional state, with fatty-acid metabolism, cholesterol trafficking, sphingolipid signaling, lipid-droplet storage, and lipid-mediator production influencing inflammatory responses, phagocytosis, efferocytosis, redox homeostasis, and tissue repair (Bamgbose et al., 2026; Zheng et al., 2026). This relationship is particularly relevant to aging, as lipid-droplet-accumulating microglia in the aged brain exhibit impaired phagocytosis, increased reactive oxygen species, and proinflammatory transcriptional programs (Marschallinger et al., 2020). Direct extrapolation of this phenotype to meningioma-associated macrophages is not warranted, however, because microglia, border-associated macrophages, and recruited monocyte-derived macrophages differ in developmental origin, tissue localization, and functional identity (Prinz et al., 2021). Nevertheless, the convergence of macrophage-rich meningioma biology with age-associated disruption of myeloid lipid homeostasis raises the testable possibility that immune-cell composition contributes to lipid states detected within meningioma tissue. Spatial co-registration of lipid imaging with macrophage identity and transcriptional state, together with lymphoid, mast-cell, and vascular markers, could determine whether specific lipid signatures localize to neoplastic or immune-rich compartments and whether these relationships vary with age or tumor progression.

### 5.4 Tumor–brain interface and invasive edge

The tumor–brain interface is anatomically and biologically distinct from the dural attachment. At this boundary, meningioma cells encounter the pia, cortical microvasculature, reactive glial populations, extracellular matrix, and, in brain-invasive tumors, the parenchyma itself. Disruption of the arachnoid–pial interface is associated with peritumoral edema and brain invasion, underscoring the structural and clinical relevance of this boundary (Zhang et al., 2026). Spatial transcriptomic profiling further demonstrates molecular heterogeneity across the tumor–brain interface: brain-invasive meningiomas exhibited alterations in cell-cycle, glycolytic, and PDGFRB-associated programs, while tumor-cell-enriched border regions showed increased KRT18, NDUFA4L2, and PKM expression, and macrophage populations varied across the tumor-to-brain axis, including increased CSF1R expression in brain-associated regions and greater infiltration in invasive tumors (Maier et al., 2026). Together, these findings support the invasive front as a spatially distinct neoplastic and immune microenvironment.

Whether lipid remodeling contributes to the transition from non-invasive tumor to brain-invasive front remains unknown. Local variation in oxygenation, metabolism, inflammatory and redox signaling, and interactions with glial and vascular cells could reshape lipid composition at this boundary. Polyunsaturated membrane lipids are particularly susceptible to oxidative modification, whereas sphingolipid and bioactive lipid-mediator pathways can regulate cellular stress, migration, vascular function, and inflammation (Cui and Ye, 2026; Farooqui and Farooqui, 2024). These mechanisms have not been demonstrated in meningioma invasion and therefore remain testable hypotheses rather than established features of the invasive phenotype. Matched sampling of tumor core, non-invasive tumor–brain boundary, and histologically confirmed invasive fronts, integrating spatial lipidomics with neuropathology, vascular and glial markers, immune-cell phenotyping, and spatial transcriptomics, could determine whether brain invasion is associated with a reproducible spatial lipid state and whether its signals arise predominantly from neoplastic cells, the cortical microenvironment, or interactions between them.

Taken together, these anatomical compartments illustrate why the meningioma lipidome should not be interpreted as a single tumor-wide state. The dural attachment, tumor–bone interface, perivascular niche, immune-rich regions, and tumor–brain boundary differ in cellular composition, vascular access, extracellular matrix organization, inflammatory exposure, and proximity to the CNS. Current lipidomic studies establish global remodeling but do not resolve this spatial architecture. Defining how individual lipid species map onto their anatomical and cellular sources is therefore a necessary next step toward understanding the functional organization of lipid signaling in meningioma. Table 2 summarizes the anatomical and cellular characteristics of these compartments, their associated or proposed lipid processes, and priorities for future spatial investigations.

**Table 2:** Anatomical compartments of the meningioma–meningeal interface: biological context, lipid processes, and spatial research priorities.

Anatomical compartment	Anatomical and cellular context	Lipid processes and molecular links	Spatial research priorities
Dural attachment	Fibroblast-rich extracellular matrix; vascular, lymphatic, and immune components; tumor–dura interactions and matrix remodeling (Wang et al., 2022)	DESI-MSI identified lipid-associated differences between tumor and adjacent dura (Calligaris et al., 2015). Inflammatory lipid mediators and lipid–matrix interactions remain candidate processes.	Compare matched tumor core, dural attachment, and uninvolved dura; relate lipid patterns to matrix remodeling and cellular composition.
Tumor–bone interface	Hyperostosis or osseous invasion in selected tumors; bone and extracellular-matrix remodeling; proximity to calvarial marrow (Mazzitelli et al., 2022; Cook et al., 2026).	Potential lipid involvement in inflammatory and osteogenic remodeling; compartment-specific lipid pathways remain untested.	Compare bone-associated tumor regions with non-bone-associated tumor tissue, adjacent dura, and bone specimens where available.
Perivascular niche	Vascular-associated NOTCH3⁺ tumor-cell populations; angiogenesis, vascular regulation, and access to circulating substrates (Choudhury et al., 2024).	Experimental meningioma models link NOTCH3 to CD36 expression, CPT1A/CPT2/HADHA induction, fatty-acid oxidation, and ferroptosis resistance (Sadagopan et al., 2025). Perivascular enrichment remains unconfirmed.	Map lipid and fatty-acid-oxidation-associated signals relative to vessels, NOTCH3⁺ tumor-cell states, and vascular or edema-related features.
Immune-rich regions	Spatially heterogeneous, macrophage-rich infiltrates; immune-cell recruitment, inflammatory signaling, and phagocytic activity (Guo et al., 2026).	Candidate cholesterol and fatty-acid handling, lipid-droplet metabolism, sphingolipid signaling, and inflammatory mediators, based partly on macrophage biology (Bamgbose et al., 2026). Their spatial role in meningioma remains untested.	Co-register spatial lipid profiles with macrophage abundance and phenotypic markers to distinguish immune- from tumor-associated signals.
Tumor core	Neoplastic-cell-rich tissue with variable vascular, stromal, and immune admixture; tumor-cell proliferation and survival.	Bulk meningioma lipidomics shows glycerophospholipid, sphingolipid, cardiolipin, and fatty-acid remodeling (Chen et al., 2026). Experimental work links FASN-mediated fatty-acid synthesis (Haase et al., 2010) and HMGCR-dependent cholesterol synthesis (Li H. et al., 2026) to PI3K/AKT signaling; spatial localization is unknown.	Determine the cellular sources of bulk lipid signals and assess spatial relationships among lipid profiles, FASN/HMGCR expression, and PI3K/AKT activity.
Tumor–brain interface	Interactions among neoplastic cells, reactive glia, vessels, and immune cells; distinct cellular programs at invasive margins (Maier et al., 2026).	Candidate membrane and polyunsaturated fatty-acid remodeling, sphingolipid signaling, and redox-sensitive lipid processes (Cui and Ye, 2026; Farooqui and Farooqui, 2024); direct links to meningioma invasion remain unproven.	Compare tumor core, non-invasive boundary, histologically confirmed invasive front, and adjacent brain, using glial and immune markers.

Lipid remodeling in meningioma has been characterized mainly in bulk tissue. FASN-, HMGCR-, and NOTCH3-associated mechanisms have experimental support in meningioma models, but their enrichment within the anatomical compartments listed here has not been established. Other compartment-specific lipid processes remain hypotheses. Aging may modify these relationships through changes in extracellular-matrix organization, meningeal lymphatic function, immune composition, and lipid homeostasis; direct spatial evidence in meningioma is lacking. ECM, extracellular matrix; FASN, fatty acid synthase; HMGCR, 3-hydroxy-3-methylglutaryl-CoA reductase; PI3K, phosphoinositide 3-kinase; AKT, protein kinase B.

## 6 Aging as a biological modifier of the meningeal lipid niche

Aging should be considered a biological modifier of the meningioma microenvironment rather than a demographic variable alone. Because meningioma incidence rises markedly with age, many tumors develop within tissues already shaped by decades of structural, vascular, immune, and metabolic remodeling. Emerging meningioma lipidomic data support the potential relevance of this context: in paired tumor–arachnoid specimens, selected lipid measures were associated with patient age, although the small cohort precludes determining whether these relationships reflect tumor-intrinsic changes, remodeling of the meninges, altered cellular composition, or systemic metabolic influences (Chen et al., 2026). Therefore, age should not yet be interpreted as a determinant of a meningioma lipid phenotype.

Structural remodeling of the aging dura could alter the meningioma microenvironment. Experimental work demonstrates age-associated peri-lymphatic collagen accumulation and increased matrix stiffness in the dura; TGF-β1 stimulates collagen production by human dural fibroblasts, while enhanced fibroblast TGF-β signaling promotes peri-lymphatic matrix deposition, disrupts lymphatic junctional integrity, impairs CSF drainage, and alters meningeal immune composition (Hitpass Romero et al., 2025). More broadly, systematic analysis of the aging brain-drainage literature indicates regionally heterogeneous remodeling of meningeal lymphatic vessels and downstream cervical lymphatic pathways, with potential consequences for fluid transport and neuroimmune homeostasis (Elyse Moore et al., 2026). In meningioma, these subtle changes could modify the local fluid exchange, immune-cell trafficking, metabolite clearance, and extracellular lipid exposure, although these effects have not been demonstrated directly.

Aging also remodels CNS lipid homeostasis, affecting cholesterol trafficking, fatty-acid metabolism, membrane-lipid turnover, lipid storage, and intercellular lipid transfer (Vanherle et al., 2025; Cerasuolo et al., 2024). In this context, astrocytes are major regulators of cholesterol synthesis, fatty-acid detoxification, lipid-droplet dynamics, and redox homeostasis, processes highly implicated in age-related and neurodegenerative dysfunction (Kim and Halliday, 2026). Similarly, lipid-droplet-accumulating microglia in the aged brain exhibit impaired phagocytosis, increased reactive oxygen species, and proinflammatory transcriptional programs, directly linking altered lipid storage with age-associated myeloid dysfunction (Marschallinger et al., 2020). Collectively, these observations provide biological context rather than direct evidence for meningioma. A central, and yet unresolved question is whether analogous age-dependent changes occur in dural fibroblasts, meningeal macrophages, vascular cells, or neoplastic cells and contribute measurably to the tumor lipidome.

Future lipidomic studies should model age as a continuous biological variable while accounting for tumor location, molecular subtype, grade, recurrence status, treatment history, sex, and cellular composition. Age-associated effects should ideally be examined across anatomically matched compartments—including tumor core, dural attachment, adjacent uninvolved dura, perivascular regions, and tumor–brain interface—rather than inferred from tumor comparisons. Integrating spatial lipidomics with histopathology and single-cell or spatial transcriptomics could distinguish tumor-intrinsic lipid changes from age-associated remodeling of stromal, vascular, and immune populations. This approach would allow testing the aging meningeal lipid niche directly as a spatial biological model, rather than inferring it from parallel observations in meningioma and CNS aging.

## 7 Toward spatial lipidomics of meningioma

Conventional lipidomics has established that meningioma lipid profiles vary with molecular genotype and histopathological grade; however, tissue homogenization removes the spatial information needed to assign lipid signals to defined anatomical or cellular compartments (Safari Yazd et al., 2023; Bogusiewicz et al., 2025). This limitation is particularly relevant in meningioma, where neoplastic cells, dura, vascular and immune populations, extracellular matrix, and brain-facing interfaces may contribute differently to the measured lipid pool. Mass spectrometry imaging (MSI) addresses this limitation by mapping lipid-associated signals within intact tissue while preserving their spatial relationship to histological architecture (Kumar, 2026; Fusar Bassini et al., 2026). Contemporary platforms based on matrix-assisted laser desorption/ionization (MALDI), desorption electrospray ionization (DESI), and secondary ion mass spectrometry (SIMS) offer complementary trade-offs in molecular coverage, sensitivity, sample preparation, and spatial resolution (Lukowski et al., 2025).

For meningioma, however, the critical methodological step precedes MSI itself: anatomical registration of tissue sampling. Surgical specimens should be oriented and sampled across predefined compartments, including the dural attachment, tumor core, perivascular or hypervascular regions, immune-rich areas, tumor–brain interface, histologically confirmed invasive front when present, and adjacent arachnoid or uninvolved dura when safely obtainable. This anatomically registered sampling strategy, including region-linked documentation and histological assessment across serial tissue sections, is illustrated in Figure 2.

**Figure 2:**
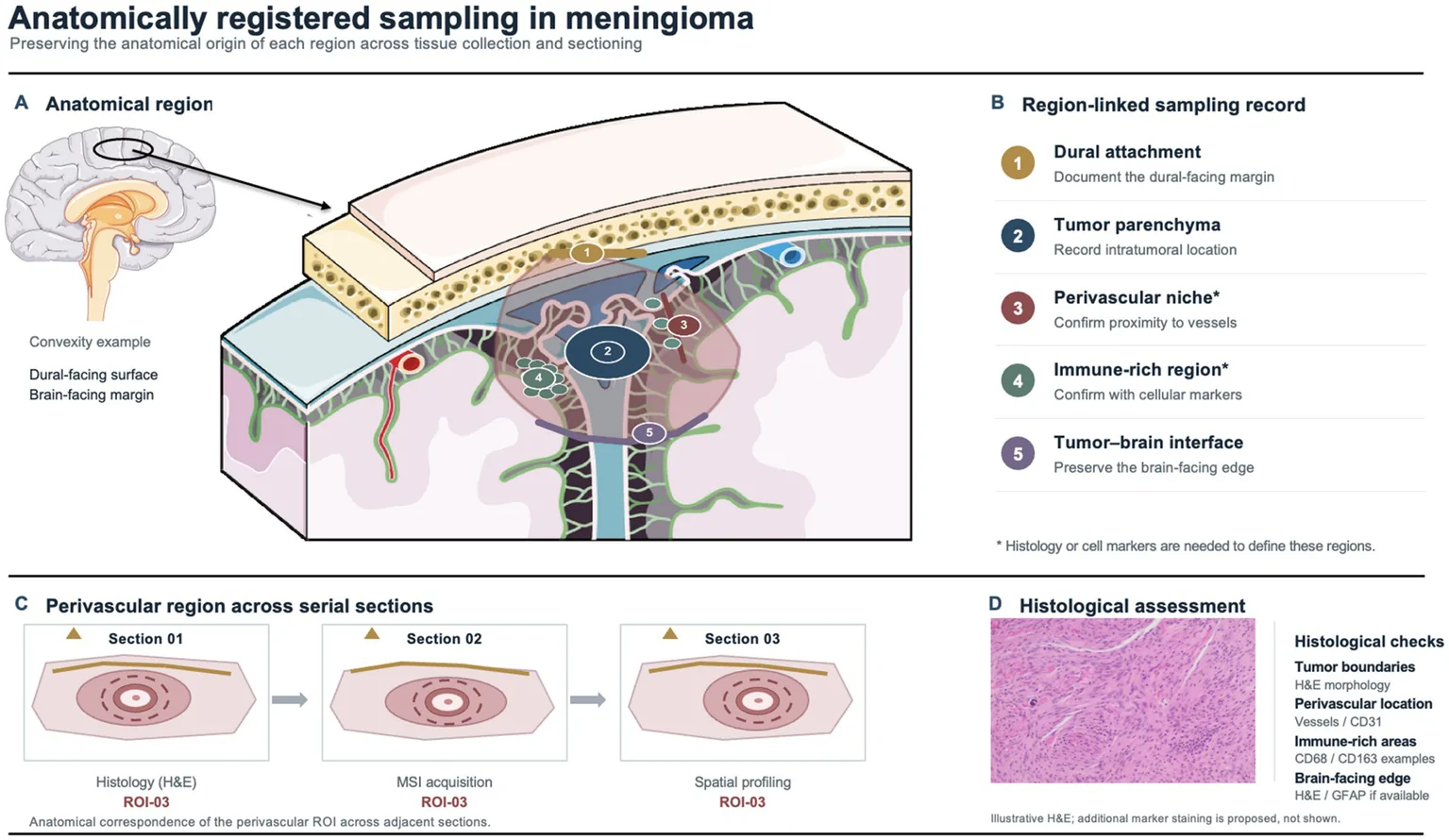
Anatomically registered sampling and histological assessment of spatial niches at the meningioma–meningeal interface. Schematic representation of an anatomically oriented sampling approach designed to preserve the spatial identity of tissue compartments during subsequent molecular analyses. **(A)** Anatomical illustration showing the relationship between the meninges, meningioma, and adjacent brain tissue. **(B)** Region-linked sampling scheme distinguishing five compartments: Dural attachment (1), tumor parenchyma (2), perivascular niche (3), immune-rich regions (4), and tumor–brain interface (5). Each region requires documentation of its anatomical origin and, where appropriate, histological confirmation. **(C)** Schematic representation of consecutive tissue sections allocated to histology, mass spectrometry imaging (MSI), and spatial profiling. The perivascular region of interest (ROI-03) is tracked across adjacent sections using a preserved dural-facing landmark, illustrating how anatomical registration facilitates the assignment of molecular measurements to corresponding tissue regions. **(D)** Representative hematoxylin and eosin (H&E) micrograph illustrating the role of histological assessment. Tumor boundaries and vascular structures may be evaluated morphologically, while additional immunohistochemical markers, such as CD31, CD68/CD163, and GFAP, may assist in identifying vascular, macrophage-rich, and glial compartments, respectively. The illustrated sampling regions and serial sections are conceptual, and the H&E micrograph is not derived from the schematic specimen. Image credits and copyright information: Anatomical illustrations adapted from Servier Medical Art (https://smart.servier.com/), licensed under CC BY 4.0 (https://creativecommons.org/licenses/by/4.0/). Representative H&E image by Mikael Häggström, obtained from Wikimedia Commons (https://commons.wikimedia.org/wiki/File:Histopathology_of_meningioma.jpg), released under CC0 1.0 (https://creativecommons.org/publicdomain/zero/1.0/).

Serial sections could then undergo histopathology, multiplex imaging, MSI, and spatial transcriptomic or proteomic profiling, allowing lipid distributions to be co-registered with neoplastic-cell density, immune-cell states, vascular architecture, extracellular matrix, proliferative regions, and molecular subtype. This strategy is feasible in meningioma: DESI-MSI has already distinguished meningioma from dura and identified lipid-associated molecular patterns related to histological subtype and NF2 aberrations (Calligaris et al., 2015).

No single MSI platform is optimal for every analytical question. MALDI-MSI offers broad molecular coverage and flexible spatial resolution and predominates in human brain spatial lipidomics, although methodological heterogeneity remains substantial (Cabasino et al., 2026). DESI requires limited sample preparation and has demonstrated feasibility in meningioma, whereas SIMS achieves higher spatial resolution but with constraints on molecular coverage and structural identification (Körber et al., 2025). These platforms are therefore complementary rather than interchangeable. Tandem MS, ion mobility, laser-capture microdissection coupled with LC–MS/MS, and targeted lipidomics can provide orthogonal structural characterization and quantitative validation of spatially localized lipid signals (Liu et al., 2026; Lukowski et al., 2025; Uchino et al., 2026).

The greatest biological value of spatial lipidomics will likely emerge from integration with complementary spatial modalities. Combining MSI with histopathology, multiplex protein imaging, laser-capture microdissection, and spatial transcriptomics can relate local lipid abundance to cellular identity and molecular state (Lukowski et al., 2025). In meningioma, such analyses could test whether phospholipid distributions co-localize with proliferative tumor regions, sphingolipid states with immune-rich compartments, cardiolipin-associated signals with mitochondrial programs, or oxidized lipids with invasive and vascular interfaces. These relationships should initially be interpreted as spatial associations rather than evidence of metabolic flux or causal signaling. Multimodal analysis could then determine whether specific lipid states are predominantly tumor-intrinsic, cell-type-associated, anatomically imposed, age-dependent, or generated through interactions among these determinants (Table 3).

**Table 3:** Multimodal framework for spatial lipid profiling of meningioma.

Modality	Primary readout	Principal strength	Key limitation	Role in proposed framework
MALDI-MSI	Spatial lipid-associated ion distributions	Broad molecular coverage with preserved tissue architecture	Ion suppression; structural ambiguity; limited absolute quantification	Discovery mapping of compartment-specific lipid states
DESI-MSI	Spatial molecular profiles	Minimal sample preparation; established feasibility in meningioma	Resolution/coverage. Trade-offs	Tumor–dura and intratumoral spatial profiling
SIMS	High-resolution molecular/fragmention distributions	Very high spatial resolution	Fragmentation and restricted molecular identification	Fine mapping of selected cellular/microanatomical interfaces
LCM–LC–MS/MS	Region-specific lipid composition	Anatomical selection with high-confidence molecular analysis	Lower throughput; predefined ROI	Orthogonal validation of MSI-defined regions
Targeted LC–MS/MS	Selected lipid species	Structural and quantitative validation	Loss of spatial information after extraction	Validation of candidate spatial lipid markers
Spatial transcriptomics	Spatial gene-expression states	Links lipid patterns to cellular and molecular programs	Expression ≠ lipid abundance or metabolic flux	Biological interpretation of spatial lipid states
Multiplex imaging	Spatial cellular phenotype	Cellular attribution of tumor, immune and vascular states	Limited direct metabolic information	Assignment of lipid signals to cellular compartments
Histopathology	Tissue architecture and pathological phenotype	Anatomical ground truth	Limited molecular information	Registration of dura, tumor, vessels, brain invasion and bone interface

DESI, desorption electrospray ionization; LCM, laser-capture microdissection; LC–MS/MS, liquid chromatography–tandem mass spectrometry; MALDI, matrix-assisted laser desorption/ionization; MSI, mass spectrometry imaging; ROI, region of interest; SIMS, secondary ion mass spectrometry. The modalities are complementary rather than interchangeable. Spatial co-localization indicates association and does not establish metabolic flux or causal signaling.

The proposed workflow, from tissue acquisition and MSI-based spatial mapping to data integration and biological interpretation, is summarized in Figure 3.

**Figure 3:**
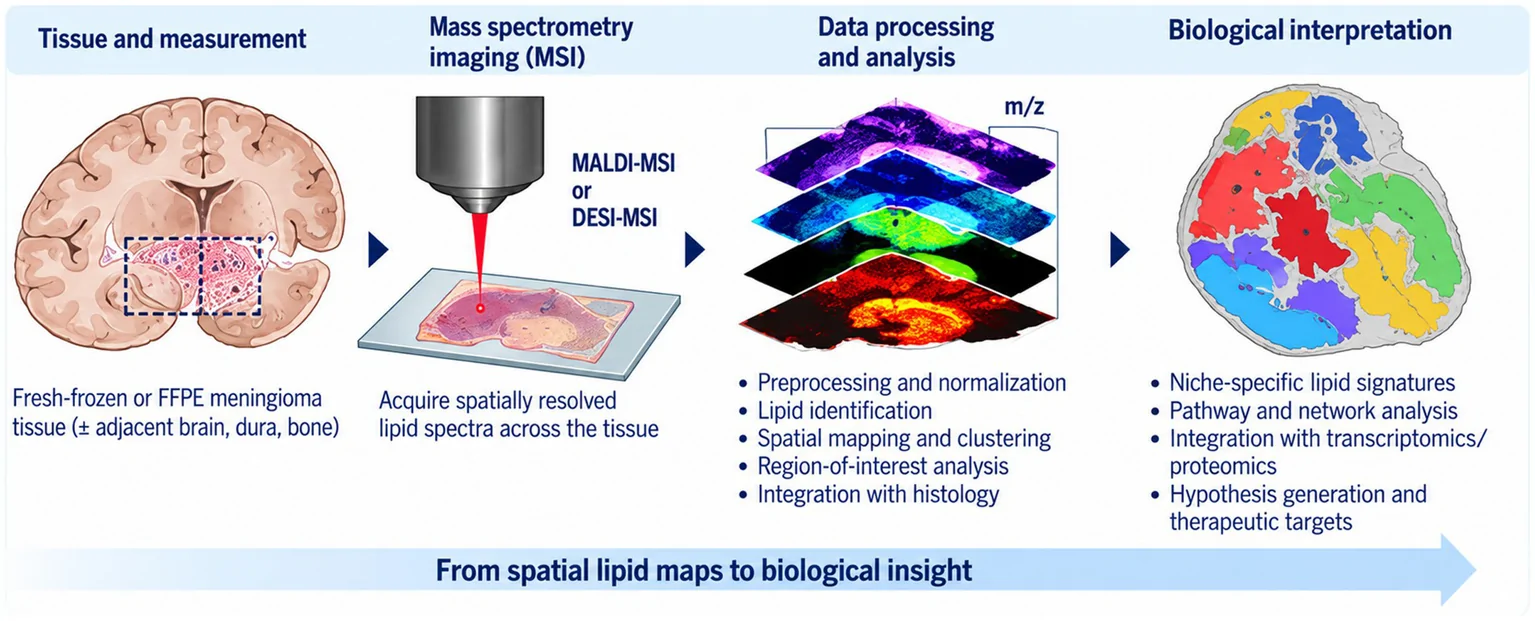
Conceptual workflow for spatial lipidomics in meningioma. Fresh-frozen or FFPE meningioma tissue can be analyzed by MALDI-MSI or DESI-MSI to generate spatially resolved lipid maps, followed by computational processing, histological integration, and biological interpretation across anatomically defined niches. Spatial associations should not be interpreted as evidence of metabolic flux or causal signaling. The figure was generated using an OpenAI generative AI tool based on scientific concepts provided by the authors and was reviewed by the authors for scientific accuracy.

Several analytical limitations must be considered in study design. Matrix effects and ion suppression can alter relative signal intensity across heterogeneous tissue regions, while lipid isomers and isobars may require tandem MS, ion mobility, or orthogonal methods for confident structural assignment; absolute quantification also remains more challenging than in extraction-based lipidomics (Liu et al., 2026). Pre-analytical factors—including tissue handling, preservation, sectioning, and matrix deposition—as well as ionization polarity and spatial resolution can further shape the detected lipid landscape (Kumar, 2026).

Spatial transcriptomics introduces additional challenges related to RNA integrity, transcript capture sensitivity, spatial resolution, and the presence of mixed-cell signals within individual capture regions (Park et al., 2023). Integrating these data with MSI also requires accurate co-registration of tissue sections and consideration of differences in spatial resolution and molecular coverage (Lukowski et al., 2025). Furthermore, transcript abundance does not necessarily reflect lipid abundance or metabolic activity, limiting direct mechanistic interpretation (Lukowski et al., 2025).

Standardized anatomical sampling and analytical workflows will therefore be essential in multicenter studies to distinguish biological spatial heterogeneity from technical variation.

A practical workflow would combine prospective anatomical annotation, serial-section histopathology, MSI-based spatial lipidomics, targeted LC–MS/MS validation, and spatial transcriptomic or single-cell reference maps. Lipid states could then be analyzed based on anatomical compartment, molecular class, grade, recurrence, invasion, edema, and patient age. This framework would directly test whether reproducible lipid states map onto anatomically and biologically distinct meningioma niches rather than representing a uniform tumor-wide metabolic phenotype.

## 8 Translational implications and future priorities

The near-term translational value of meningioma lipid biology lies in biological stratification and spatial phenotyping rather than therapeutic targeting. Current human lipidomic studies demonstrate associations between lipid composition, molecular genotype, and histopathological grade, while experimental evidence implicates FASN, HMGCR, and NOTCH3-associated fatty acid oxidation as potential metabolic targets. However, these mechanistic findings remain preclinical, and no lipid-directed therapeutic strategy has been clinically validated in meningioma.

Lipid states should therefore be evaluated as candidate correlates of clinically relevant features, including recurrence, brain invasion, peritumoral edema, bone involvement, and treatment resistance. The perivascular NOTCH3-positive compartment provides a relevant model, representing a spatially defined tumor-cell state that promotes tumorigenesis and resistance to radiotherapy (Choudhury et al., 2024). Determining whether such compartments harbor specific lipid dependencies could reveal metabolic vulnerabilities, but therapeutic translation will require functional validation.

A major practical priority is anatomically informed tissue collection. Labeling a surgical specimen simply as “meningioma” obscures its compartment of origin. Prospective biobanking should therefore preserve tissue orientation and distinguish, where feasible, tumor core, dural attachment, tumor–bone interface, vascular regions, tumor–brain boundary, and invasive front. Linking these compartments to histopathology, imaging features, and molecular classification would improve the interpretability of lipidomic and other multi-omic datasets. Integrating edema, bone involvement, vascularity, and anatomical location with molecular and lipidomic data may further capture biologically relevant heterogeneity obscured by analyses centered on histopathological grade alone.

Several priorities emerge from current evidence. Meningioma lipidomics requires validation in larger, molecularly classified cohorts, while the cellular origins of altered lipid signals and their relationships with immune, vascular, and stromal states remain unresolved. The dural attachment, tumor–bone interface, perivascular niche, immune-enriched regions, and invasive front should be profiled as distinct compartments, with age incorporated as a continuous variable. These gaps generate testable hypotheses: invasive fronts may exhibit lipid states distinct from tumor cores; dural attachments may show lipid profiles associated with matrix and vascular remodeling; and macrophage-rich regions may harbor distinct lipid-storage or lipid-mediator programs. Anatomical location should also be incorporated because genotype varies across meningioma sites, with NF2 alterations enriched in convexity tumors and non-NF2 alterations at the skull base, and finer genotype–location relationships reported within the foramen magnum (Ali et al., 2025; Hua et al., 2024). Testing these hypotheses in spatially registered, molecularly classified cohorts should take priority over descriptive profiling of bulk tumor tissue.

## 9 Conclusion

Meningioma lipid biology is emerging alongside major advances in meningeal anatomy, molecular classification, and spatial profiling. Current evidence demonstrates substantial lipid remodeling but does not support a uniform meningioma lipid phenotype. We propose the aging meningeal lipid niche as a spatial framework linking lipid signaling across the dura, tumor–bone interface, vasculature, immune microenvironment, tumor core, and tumor–brain boundary. Testing this model will require anatomically registered sampling integrated with spatial lipidomics, histopathology, molecular classification, and single-cell or spatial transcriptomic approaches. Such studies could determine how aging, anatomical context, and cellular composition shape meningioma lipid states and their relationships with invasion, recurrence, edema, and treatment resistance. Ultimately, this framework positions lipid biology as a spatially organized component of meningioma pathophysiology, linking tumor-intrinsic programs with the aging meningeal tissue microenvironment.
